# Bile—Its Offices in Health, Abnormal Effects, &c.; a Thesise Presented to the Faculty of the Atlanta Medical College

**Published:** 1859-10

**Authors:** J. Emmett Blackshear

**Affiliations:** Marietta, Georgia


### ARTICLE III.

*Bile—Its Offices in Health, Abnormal effects, &c.; A Thesese Presented to the Faculty of the Atlanta Medical College.* By J. EMMETT BLACKSHEAR, Marietta, Georgia. Session 1859.

There is, perhaps, no secretion of the human body that has attracted more attention than the bile, and none, I opine, the offices, abnormal conditions and effects of which, are not more thoroughly understood, more accurately defined. It is exceedingly common to hear persons, both professional and non-professional, speak of being bilious, of derangement of the biliary secretion, &c., and often has the liver been made to pay the severe penalty of disease in organs wholly unconnected with this secretion. Some are in the habit of referring almost every febrile effection to derangement of this organ, and deem their hypothesis sufficiently substantiated by the fact that when emesis occurs, either from the administration of emetics or otherwise, bile is sometimes seen mingled with the ejection. In this case, the patient is supposed to be *bilious*. When no bile occurs the derangement is attributed to *an insufficient secretion of this fluid.*

That derangement in this, as well as all the other secretions, is almost invariably the case in fever, is undoubtedly true, but I am fully persuaded that too many are in the habit of confounding cause with effect. Now, while we are willing to admit, that derangements in the functions of the liver are often fruitful sources of disease, we cannot see any good reason why this organ, while in an unoffending state, should be so frequently and severely scourged, simply because of the incompetency of the physician to diagnosticate, or trace the true pathology of the disease which he is called upon to treat.

The bile has been so long considered an indispensable agent in digestion, that, to question its utility in this respect, would seem almost like attacking an *aneient landmark* of the science, yet, when we observe minutely the changes that occur in digestion, we are struck with astonishment to find, that this important process may be carried on, *a capite ad calcino,* without calling into requisition, this hepatic secretion.

If we observe the food in the various stages of digestion, we shall see it acted upon, and disposed of in the following manner: During the act of mastication, it is mingled with the saliva, and prepared for deglutition by being formed into a pulp or bolus, in which condition it passes through the œsophagus into the stomach. By the muscular action of this organ, it is tossed to and fro, and thoroughly mingled with the gastric juice, the active flow of which is excited by the presence of food in the stomach. By these processes it is changed into a pultaceous substance called chyme—the albumen, gelatine, fibrin and caseine being converted into a low form of albumen called albuminose or peptone, starch and lignine, into grape sugar, and the oleaginous principles simply reduced to minute particles. In this mixed condition, the whole passes through the piloric orifice of the stomach, into the duodenum, where, coming in contact with the pancreatic juice, the fatty matters are digested, or reduced to the state of an Emnlsion, and the whole converted into chyle, which is taken up by the Lacteals, carried through the mesenteric glands, to the thoraeic duct, and finally poured into the left subclavian vein, and carried through the circula tion to all parts of the system, to administer to the nutrition of the body.

Now, during the process of digestion, the secretion of bile is very active, and as its flow into the duodenum is at that time greatly accelerated, we would very naturally suppose that it was intended to perform some important part in the formation of chyle; but what office it here fills, or whether any at all, are questions which Physiology, in the present state of the science, is unable to answer. It was for a long time considered the active agent in the digestion of the oleaginous particles of the food, an office which is now assigned to the pancreatic fluid. In corroboration of other reasons for adopting this latter opinion, I would simply mention the fact, that in animals that take large quantities of oleaginous food, the pancreas is proportionably large, while the converse is true of those which take small quantities of this material.

Then, while we would not have the arrogance to declare that it fills no indication, we would very modestly assert, that we have sought in vain for the office of bile in digestion. It is contended by some, that, by admixture with the chyme, it prevents destrutive chemical change in its composition; but it has been proven by experiment on animals, that the digestive function can be carried on naturally, even though all the bile secreted be discharged from the gall bladder, out of the body, without ever coming in contact with the food.

Let us then enquire, what is the office of this extensive secretion? We know that bile is always (in health) mingled with the faeces; from which circumstance it has been called the natural purgative of the body. That—perhaps by promoting an increased secretion of the intestinal glands, &c.,—it performs an office of this kind, is evidently true; but the proportion voided in this way is very small in comparison with the amount secreted, not more, it is said, than "1-16th part of its weight, including only its coloring and some of its fatty matters, and none of the essential principles, the biline." What then becomes of the residue? It must be absorbed from the intestines, and again taken into the circulation; but from its elementary composition we know that it cannot contribute to the nutrition of the tissues of the body. Yet we know that it is disposed of in some way, else its accumulation in the system would soon produce disease and death. We are led to the conclusion then, inasmuch as it is made up principally of these elements which are the most combustible, viz: Carbon and Hydrogen, that, coming in contact with the Oxygen of the body, it is burned for the purpose of assisting in the maintenance of the animal temperature, and finally thrown off at the lungs, in the form of carbonic acid and water. This is doubtless the office filled by the re-absorbed portion of the bile. Nor is it an unimportant one, for, in the experiment of Blondolt, above mentioned, where the bile was discharged from the gall bladder, by means of a fistulous opening from the surface, it was found that tho' digestion went on uninterruptedly, yet the animal, in a few months, "died with inanition; such signs, it may be supposed, as would be produced by the deficiency of Carbon and Hydrogen in the blood."

Now, during the period of intra-uterine existence, there is no need for the consumption of the bile as fuel, the animal temperature of the fœtus being maintained by that of the mother, through the placenta. It is, therefore, highly probable that, instead of being re-absorbed, all the bile secreted during that period is incorporated with the meconium, (which analysis shows to contain all its essential principles,) and directly excreted. The blood being, through this process, purified by the direct excretion of carbon and hydrogen, in the same manner that it is an extra uterine existence, by the throwing off of carbonic acid and water at the lungs, the liver is truly said to perform an office in the fœtus vicarious of that of the lungs.

Abnormal conditions of this secretion, as before stated, are not unfrequently the cause as well as effect of disease.

Like all the other secretions, it has its own peculiar offices to fill, an interruption of which soon throws the system into disorder. When we consider the size, situation, extensive anatomical relations, excitability, and various functions of the liver, we are not surpised at its frequent derangements—which always tends to increase or diminish its secretive power—and the general disorder which is the invariable result.

Of the causes that produce these derangements of the liver—and consequently, abnormal conditions of the bile—there are none, perhaps, more common than atmospheric heat and malaria, the effects of which are to stimulate the organs to over-exertion. From this undue excitement, the powers of the liver soon become exhausted; to relieve which, the vital forces, forsaking the parts to which they are normally distributed, become concentrated in this enfeebled organ. The capillaries, from the withdrawal of the nervous power from them, become relaxed. The arteries, therefore, immediately empty themselves into the veins, and as the flow of blood through the lungs is retarded by the loss of power in the organs of respiration, venous engorgement, with all the phenomena of the cold or congestive stage of chill, is the inevitable result. This is the opening scene of what is generally known as *bilious fever*. When this stage of depression has existed for an indefinite period, the recuperative powers of the system are again brought into requisition. Re-action takes place. The nervous force is redistributed to the parts from which it was withdrawn. The organs of respiration being thus relieved, the blood again flows freely through the lungs, but being retarded in its progress through the capillaries—they having regained their tonicity—an opposite state of things is produced, viz: arterial engorgement, with all the phenomena of fever. This state of excitement gradually subsides, the balance in the circulation is restored, and the paroxysm is brought to an end. In all this disturbance, it is very evident that the superfluity of bile is more a symptom than a cause of disease.

*An excess of bile* is sometimes, however, the direct excitant of disease; for instance, a greater quantity than usual coming in contact with the mucus membrane of the intestines, frequently produces irritation, which not uncommonly ends in diarrhœa. In this instance, there is—particularly if there be depravity of the bile, which is most commonly the case—violent gastric and intestinal pain, and sometimes spasm. The alvine evacuations are of an unnatural color, generally brown or black, and sometimes of a tarry consistence. When the affection is aggravated and vomiting ensues, it constitutes cholera morbus. Diarrhœa is sometimes caused, however, by an insufficient secretion of bile, which is marked by light-colored evacuations. Again, the reflux of bile into the stomach is said to check the process of fermentation, thereby interfering with gastric digestion; and if we adopt the opinion that its admixture with chyme prevents destructive chemical action, we must acknowledge that when an insufficient supply is furnished, abnormal putrefaction must be the result.

Colic is another disease dependent on an excess and vitiated state of this secretion, in which there is spasm of the intestine. "The immediate cause of the spasm," says Professor Wood, "is probably an irritation of the mucous coat of the bowels, induced either by sympathy with irritation of the liver, or by a congested state of the portal veins, or by both."

*Deficiency of secretion*, dependent either upon a diminished quantity of material in the blood, for the formation of bile, or functional derangement of the liver, is apt to produce constipation, with its characteristic symptoms, some of which are as follows: anorexia, depression of spirits, with general disquietude, irritability of temper, flushed face, cephalalgia, dyspnœa, drowsiness, disordered vision, &c. Now, regarding the bile as the natural purgative of the body, by which the bowels are lubricated, and excited to that peculiar peristaltic action which enables them to free themselves of the efete materials that are constantly thrown into them, we can readily understand how the withdrawal of this stimulus should produce this constipation. And, bearing in mind the close relation that exists between the primaviæ, &c., and the brain, we are not at a loss to account for the symptoms enumerated.

When *functional derangement* of the liver is the cause of the diminished or suspended secretion, we are apt to have that yellowness of skin characteristic of Jaundice. The incapacity of the liver to eliminate from the blood, the poisonous materials which enter into the formation of bile, would be productive of the most serious consequences, were it not for the wise provision of nature, made in "rendering almost all the other secreting functions sensible to the stimulus of the yellow materials in excess, and capable, under this stimulus, of separating it from the blood." It is by this substituted process of elimination, through the skin and kidneys, that the coloring matter of bile becomes mingled with the tissues, imparting to the skin, eyes, &c., the peculiar tinge above mentioned.

The bile is subject to various morbid changes in disease; for instance, in extensive inflammation, and in some cases of typhus and dropsy, it is said to be more watery. In diseases of the heart, affections which cause congestion of the large veins, the solid contents are said to be increased. In malignant cholera, it is said to be very thick and tenacious.

The two conditions which are favorable to gall stones, viz.: a concentration of the coloring matters, and an excess of cholesterine, are deserving of notice, but time and space forbid.

